# Desert Dust as a Source of Iron to the Globally Important Diazotroph *Trichodesmium*

**DOI:** 10.3389/fmicb.2017.02683

**Published:** 2018-01-17

**Authors:** Despo Polyviou, Alison J. Baylay, Andrew Hitchcock, Julie Robidart, C. M. Moore, Thomas S. Bibby

**Affiliations:** ^1^Ocean and Earth Science, University of Southampton, Waterfront Campus, Southampton, United Kingdom; ^2^Department of Molecular Biology and Biotechnology, University of Sheffield, Firth Court, Sheffield, United Kingdom; ^3^Ocean Technology and Engineering Group, National Oceanography Centre, Southampton, United Kingdom

**Keywords:** *Trichodesmium*, iron, dust, nitrogen fixation, cyanobacteria

## Abstract

The marine cyanobacterium *Trichodesmium* sp. accounts for approximately half of the annual ‘new’ nitrogen introduced to the global ocean but its biogeography and activity is often limited by the availability of iron (Fe). A major source of Fe to the open ocean is Aeolian dust deposition in which Fe is largely comprised of particles with reduced bioavailability over soluble forms of Fe. We report that *Trichodesmium erythraeum IMS101* has improved growth rate and photosynthetic physiology and down-regulates Fe-stress biomarker genes when cells are grown in the direct vicinity of, rather than physically separated from, Saharan dust particles as the sole source of Fe. These findings suggest that availability of non-soluble forms of dust-associated Fe may depend on cell contact. Transcriptomic analysis further reveals unique profiles of gene expression in all tested conditions, implying that *Trichodesmium* has distinct molecular signatures related to acquisition of Fe from different sources. *Trichodesmium* thus appears to be capable of employing specific mechanisms to access Fe from complex sources in oceanic systems, helping to explain its role as a key microbe in global biogeochemical cycles.

## Introduction

Cyanobacterial diazotrophs are responsible for most of the fixed-nitrogen entering marine ecosystems and as such have a major role in regulating oceanic productivity (Mahaffey et al., 2005; Zehr and Bombar, 2015). The availability of iron (Fe) in marine ecosystems heavily regulates the biogeography and activity of such cyanobacteria (Mills et al., 2004; Moore et al., 2009; Chappell et al., 2012; Snow et al., 2015b) due to the absolute requirement of Fe in the catalysts of both photosynthesis and dinitrogen (N_2_) fixation (Geider and La Roche, 1994; Sañudo-Wilhelmy et al., 2001; Shi et al., 2007; Richier et al., 2012). The colonial, non-heterocystous cyanobacteria *Trichodesmium* sp. are responsible for almost half of the N_2_ fixed in marine systems annually (Capone et al., 1997; Mahaffey et al., 2005). *Trichodesmium’s* requirement for Fe is thought to be enhanced relative to single-celled or heterocystous species as it simultaneously requires Fe for both photosynthesis and N_2_ fixation (Berman-Frank et al., 2007), processes which are temporally or spatially separated in most N_2_ fixing microorganisms (Tuit et al., 2004). *Trichodesmium* may therefore need to be particularly adept at acquiring Fe from the environment.

Desert dust is a major source of Fe to the surface ocean (Jickells et al., 2005). Total Fe supply from dust is dominated by particulate (>0.4 μm) or colloidal (0.02–0.4 μm) rather than readily soluble (<0.02 μm) forms (Aguilar-Islas et al., 2010; Fitzsimmons et al., 2015), although solubility of Fe from dust can vary further as a function of either dry or wet deposition modes and the time spent interacting with a variety of processes occurring within the oceanic mixed layer (Croot et al., 2004; Baker and Croot, 2010; Schlosser et al., 2014). Comparisons between computations of the total dust inputs and the Fe inventory of the oceans indicate low overall solubility of Fe from dust (<12%) (Jickells and Spokes, 2001). Although soluble Fe is typically considered to be the major source of bioavailable Fe to phytoplankton (Wells, 1989; Schlosser and Croot, 2008; Chester, 2009; Fitzsimmons, 2013) particulate/colloidal forms have been suggested to play a role (Kuma and Matsunaga, 1995) and a growing number of studies have demonstrated that microbes can access Fe from these sources (Nodwell and Price, 2001; Frew et al., 2006; Sugie et al., 2013; van der Merwe et al., 2015), with *Trichodesmium* potentially capable of accessing Fe from both colloidal (Wang and Dei, 2003) and particulate (Rubin et al., 2011) forms.

Cyanobacteria display a number of distinct Fe acquisition pathways including Fe^3+^ and Fe^2+^ transporters, the latter often coupled to biological reduction of Fe^3+^ to Fe^2+^, alongside production of siderophores which are released from the cell, bind Fe and are subsequently taken up through dedicated transporters (Neilands, 1995; Hopkinson and Barbeau, 2012; Boiteau and Repeta, 2015). Homologs to Fe^2+^ (FeoAB), Fe^3+^ (FutABC) and siderophore (FhuD) transporter components are encoded in the *Trichodesmium* genome; however, the proteins involved in Fe reduction are not well characterized (Chappell and Webb, 2010). Inorganic Fe reduction and uptake is possibly facilitated by reactive oxygen species (ROS) produced intracellularly (Roe and Barbeau, 2014; Hansel et al., 2016) while cell surface processes appear to be involved in observed facilitated dissolution of Fe from dust by *Trichodesmium* (Rubin et al., 2011). These include active trapping and directed transport of particulate Fe into the core of *Trichodesmium* colonies (Rubin et al., 2011). However, the mechanisms and physiological impacts of cell-to-substrate contact for acquisition of Fe from dust are yet to be fully determined.

Using non-axenic *Trichodesmium erythraeum* IMS101 (*Trichodesmium*) cultures, we investigate the Fe uptake strategies involved in accessing Fe from Saharan desert dust within an experimental situation. We evaluate the significance of permitting cell contact for acquiring Fe from dust by physically separating dust from cells using porous membranes. We report both on the physiological and transcriptomic responses of *Trichodesmium* to bound (particulate and colloidal) and free dissolved Fe to understand the factors that enable this key microbe to apparently dominate N_2_ fixation within regions of high dust input.

## Materials and Methods

### Culture Conditions and Growth

*Trichodesmium erythraeum* IMS101 (*Trichodesmium*) was grown on an orbital shaker (150 rpm) under a 12h dark: 12h light cycle (ca. 130 μmol photons m^-2^ s^-1^) in modified YBC-II medium (Polyviou et al., 2015; Snow et al., 2015a) having a final EDTA concentration of 20 μM (Hong et al., 2017). Experimental cultures at a total volume of 40 ml were initiated from an Fe replete stock culture rinsed with no-added Fe media before inoculation and incubation in triplicate under 4 conditions: (Fe-) with no added Fe, (Fe+) with 400 nM added FeCl_3_-EDTA as a source of dissolved soluble Fe (sFe), equivalent to a calculated bioavailable inorganic Fe concentration of 1100 pM Fe’ (Snow et al., 2015a) and two treatments with 0.25 mg ml^-1^ of Saharan dust, collected between 04°43’N, 28°55’W and 06°56’N, 28°07’W using a mesh system aboard the RSS Shackleton (Murphy, 1985). Dust was added either directly (Dust+) or inside a barrier to the culture created using 8 kDa MWCO dialysis tubing (DT) (BioDesign, Carmel, NY, United States) ([Dust]). For consistency and to confirm that diffusion of sFe through the membrane was possible the FeCl_3_-EDTA was also released into the Fe+ media through the DT. DT was washed by boiling in 120 mM Na_2_HCO_3_ and subsequently in 10 mM Na_2_EDTA and 10 mM NaOH twice before rinsing three times in milli-Q H_2_O. Clean DT, handled with 10% HCl washed plastic tweezers, was included in all experimental treatments except for a no-DT control. The no-DT control was grown under no added Fe conditions (i.e., as per the Fe- treatments) with the physiology of *Trichodesmium* showing no significant differences when compared to the Fe- treatment, suggesting that inclusion of DT did not result in any significant Fe contamination (Supplementary Figure S1). Within a separate control experiment the physiological responses of *Trichodesmium* were monitored to test whether nutrients from dust dissolved preferentially when dust was free in the culture vessels compared to when it was enclosed in DT (Supplementary Figure S2). Within this control experiment, the same series of treatments (i.e., +Fe, -Fe, Dust+ and [Dust]) were incubated abiotically, i.e., without the addition of *Trichodesmium* for a period of 14 days before filtration through a 0.2 μm filter into clean culture flasks and subsequent inoculation of the filtrate (i.e., with the dust particles removed) with equal concentrations of cells.

*Trichodesmium* growth was monitored by cell counts performed using a Sedgewick Rafter counting chamber and a GX CAM-1.3 camera on an L1000A biological microscope (GT Vision Ltd., Suffolk, United Kingdom). Cell and filament lengths were identified using GX capture (GX 14 Optical, Suffolk, United Kingdom) and ImageJ (Schneider et al., 2012). The concentration of cells was calculated for the duration of the experiment (Supplementary Figure S3) by dividing the total filament length by the average cell length and growth rates calculated using the gradient of the natural logarithm of measurements during exponential growth.

### Photosynthetic Physiology

Photosynthetic physiology was monitored using a FASTtracka^TM^ MkII Fast Repetition Rate fluorometer (FRRf) integrated with a FastAct^TM^ Laboratory system (Chelsea Technologies Group Ltd., Surrey, United Kingdom). Measurements were made 2.5 h after the beginning of the photoperiod following dark adaptation of samples for 20 min and consequent exposure to a background irradiance of 29 μmol photons m^-2^ s^-1^ for 2–5 min (Richier et al., 2012). *F*_v_/*F*_m_ was used as an estimate of the apparent PSII photochemical quantum efficiency (Kolber et al., 1998). Data presented is the average of three technical replicate measurements for each of the three biological replicates.

### RNA Sequencing

*Trichodesmium* cultures were filtered onto GF/F filters on day 17 of the experiment and snap frozen in liquid nitrogen. RNA was subsequently extracted using the RNeasy Plant Mini Kit (Qiagen, Manchester, United Kingdom), treated with TURBO DNA-free^TM^ DNase (Life Technologies Ltd., Paisley, United Kingdom) and stored at -80°C. Absence of genomic DNA contamination was confirmed by PCR.

Paired end libraries for Illumina sequencing were prepared using the TruSeq Stranded mRNA Library Prep Kit (Illumina), following the manufacturer’s low-throughput protocol. This was modified for use with bacterial RNA samples by replacing the initial poly-A-based mRNA isolation step with ribosomal RNA (rRNA) depletion using a bacterial Ribo-Zero rRNA Removal Kit (Illumina Inc., San Diego, CA, United States). Libraries were pooled and sequenced on an Illumina MiSeq instrument using paired-end sequencing, with a read length of 151 bp.

The raw sequence reads were pre-processed with CutAdapt (v1.8.1, Martin, 2011) to remove TruSeq adapter sequences and low quality bases from the 3′ end of reads, using a quality threshold of 15 and minimum read length of 36 bp. Trimmed reads were mapped against the *Trichodesmium erythraeum* IMS101 reference genome assembly (Ensembl Bacteria database, accession GCA_000014265.1) using TopHat v2.0.14 (Kim et al., 2013), and reads mapping to annotated genes in IMS101 were counted using HTSeq, version 0.6.0, (Anders et al., 2015). The –library-type fr-firststrand and -stranded = reverse options were used in TopHat and HTSeq, respectively, to correctly account for strand-specific read mapping. Libraries were normalized based on the total library size using the median-of-ratios method that is implemented in DESeq2 (Love et al., 2014).

### Differential Gene Expression Analysis

Differential gene expression analysis was carried out using the Bioconductor package DESeq2 version 1.8.2 (Love et al., 2014), running on R version 3.2.0. A set of genes with expression significantly affected by growth condition was identified using an ANODEV approach implemented in the DESeq2 package. The fit of read count data against a one-factor negative binomial general linear model, where replicates were grouped by growth condition, was compared (using likelihood ratio testing) to a reduced model where condition information was removed. Pairwise contrasts between conditions were calculated using a Wald test. In both cases, genes with an adjusted *P*-value of < 0.05 following Benjamini–Hochberg correction for multiple testing were classed as significantly differentially expressed.

The results of the RNA sequencing (RNAseq) analysis were validated via quantitative reverse transcription (RT) Polymerase Chain Reaction (qPCR) using the cytochrome c_553_ gene (*cyt c_553_, Tery_2561*) the iron-stressed-induced protein-A gene (*isiA, Tery_1667*) and the Mo-dependent nitrogenase-like-protein gene (nif-like, *Tery_4114*). Patterns of expression from the two methods closely resembled each other and regression analysis indicated a good correlation (*R*^2^ = 99%) (Supplementary Figure S4).

### Hierarchical Clustering

To group differentially transcribed genes by transcription profile across treatments, the Pearson product moment correlation coefficient (PPMCC) was calculated for each pair of genes from regularized log (rlog) transformed read count data. Hierarchical clustering was then carried out using the WPGMA method, with 1-PPMCC as the distance metric. Similarly, to cluster samples based on overall transcription profile, pairwise sample distances were calculated using correlation coefficients and hierarchical clustering was carried out using the average linkage method (Minitab 17.3.1, Minitab Inc., Coventry, United Kingdom).

## Results

### Effects of Desert Dust and Fe’ on *Trichodesmium* Physiology

**FIGURE 1:**
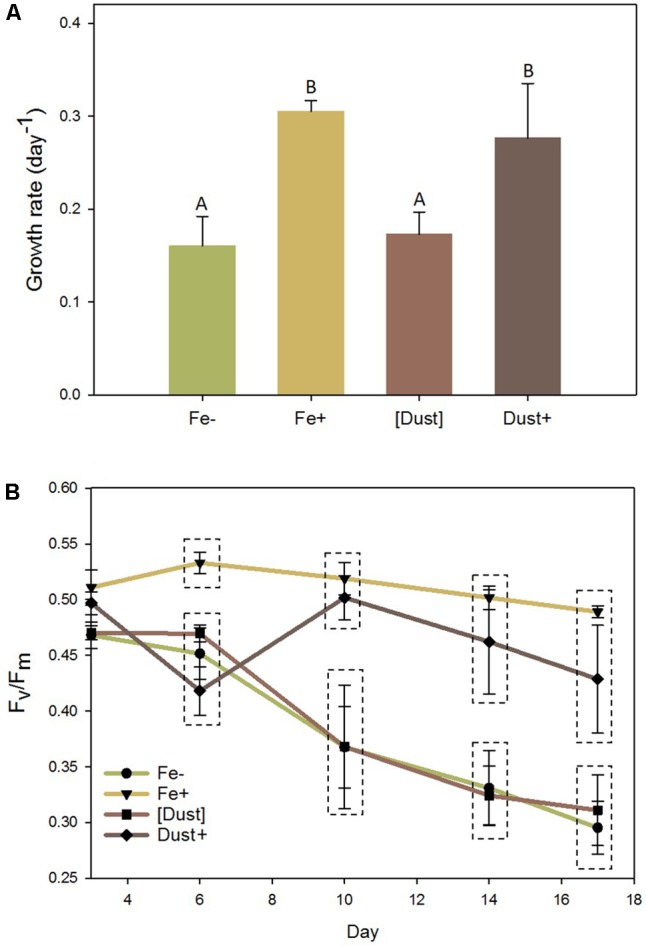
Effects of desert dust and Fe’ on *Trichodesmium* physiology. Dust added (Dust+, dark brown), dust separated ([Dust], light brown), Fe’ replete (Fe+, yellow) and Fe’ deficient (Fe–, green) *Trichodesmium* cultures were monitored over 17 days during which **(A)** growth rates (day^-1^) and **(B)** photosynthetic efficiency (*F*_v_/*F*_m_) were recorded. Statistically significant differences (GLM and Tukey test; *P* < 0.05) between treatments are indicated with dissimilar letter groups **(A)** or dashed boxes **(B)**. Error bars represent standard deviations of three biological replicates. (GLM and Tukey test, *P* < 0.05).

The effect of Saharan desert dust addition on the physiology of non-axenic *Trichodesmium erythraeum IMS101* (hereafter *Trichodesmium*) when in the direct vicinity (Dust+) or separated ([Dust]) from the cells within otherwise low Fe medium was compared to iron deplete (Fe-) and iron replete (Fe+) treatments (as described in Materials and Methods). Growth of *Trichodesmium* was significantly faster under Fe+ (0.31 day^-1^) and Dust+ (0.28 day^-1^) compared to Fe- (0.16 day^-1^) and [Dust] (0.17 day^-1^) conditions (**Figure 1A** and Supplementary Figure S3) (GLM and Tukey test; *P* < 0.05). Additionally, while photosynthetic efficiency (*F*_v_/*F*_m_) declined rapidly in Fe- and [Dust] treatments from day 6 until the end of the experiment, it was maintained at significantly higher levels in Fe+ and Dust+ treatments (**Figure 1B**). In contrast, although the Fe+ media similarly supported a higher growth rate within the abiotic control experiment, growth rates within media preincubated with dust either inside or outside the DT were statistically indistinguishable from both each other and the -Fe treatment (Supplementary Figure S2).

### Transcriptomic Response of *Trichodesmium* to Desert Dust and Fe’

**FIGURE 2:**
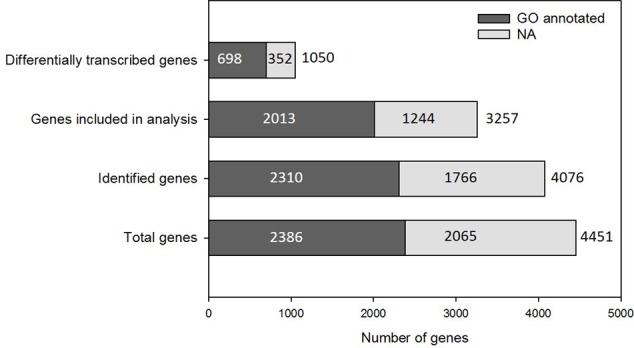
Gene numbers and GO annotation coverage. The majority of *Trichodesmium* genes are identified in at least one condition of the experiment. Genes for which expression is recorded in all treatments above threshold quantities are analyzed to identify differential transcription. A large proportion of the *Trichodesmium* genome is not GO annotated (NA, light gray).

Samples collected on the last day of the main experiment (day 17) were analyzed using RNAseq to ascertain the gene transcription profiles relating to each experimental condition. The *Trichodesmium erythraeum* IMS101 genome is composed of 4451 annotated genes, ∼54% of which are annotated with a Gene Ontology (GO) classification number (GO annotated) (**Figure 2**). The majority of *Trichodesmium* genes (∼98%) were identified (read counts/million > 0) to be transcribed in at least one treatment and 4076 (∼92%) were identified in all four treatments. Of these, 3257 genes had read counts above the statistical threshold required for inclusion in differential gene transcription analysis, as determined by the DESeq2 Bioconductor package (Love et al., 2014). Approximately one third of those genes (1050) were significantly affected by growth condition (adjusted *P*-value < 0.05 following Benjamini–Hochberg correction for multiple testing, DESeq ANODEV) (**Figure 2**).

**FIGURE 3:**
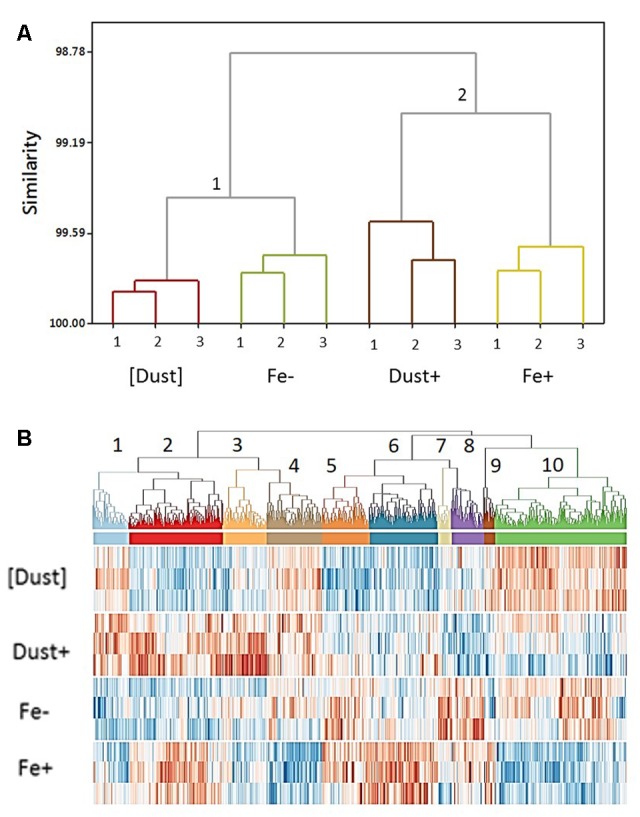
**(A)** Clustering of gene transcription across treatments and replicates. Replicates of each treatment ([Dust], Fe–, Dust+, Fe+) cluster close together while transcription in (1) [Dust] (light brown) and Fe– (green) cultures groups closer together compared to (2) Dust+ (dark brown) and Fe+ (yellow) cultures. To calculate distances the average linkage method was used. **(B)** Clustering of transcription across all differentially regulated genes. Ten clusters of different transcription profiles across the [Dust], Dust+, Fe–, and Fe+ treatments (computed using the Pearson product moment correlation coefficient between each pair of differentially expressed genes and WPGMA) are detailed in a heat map of transcript level in a dendrogram.

The validity of experimental biological replicates was confirmed through hierarchical clustering of gene transcription profiles which revealed closer intra-treatment compared to inter-treatment similarities (**Figure 3**). In addition, two distinct groups of treatments were resolved, highlighting similarities in gene regulation between [Dust] and Fe- treatments (group 1) and between Fe+ and Dust+ treatments (group 2) (**Figure 3A**).

A heat map illustrating all 1050 differentially regulated genes identified in the transcriptomic analysis revealed 10 distinct patterns of gene transcription from the clustering analysis (**Figure 3B** and Supplementary Figure S5) demonstrating significant changes in gene expression across all treatments. The majority of genes (42%) fall in clusters regulated similarly between Fe+ and Dust+ treatments with either increased (Cluster 2, *n* = 185) or reduced (Cluster 10, *n* = 259) transcription relative to Fe- and [Dust] (Supplementary Figure S5). Differential gene regulation in Fe+ compared to Fe-, Dust+ and [Dust] treatments (Supplementary Figure S5) is also observed for a large number of genes (Cluster 4, *n* = 109 and Cluster 6, *n* = 135).

### Known Markers of Iron Limitation

The expression of a set of 12 genes (Supplementary Table S1) of known importance during Fe stress conditions and/or previously identified to be differentially expressed (at the transcript or protein level) under variable Fe concentrations was examined in order to access the molecular response to Fe specifically. The genes include the chlorophyll-binding antenna (*isiA*, iron stress induced protein A gene, *Tery_1667*) (Bibby et al., 2001; Shi et al., 2007; Richier et al., 2012; Snow et al., 2015a), the flavodoxin genes (*fld1, Tery_1666*; *fld2, Tery_2559*) which are an Fe free alternative to ferredoxin (LaRoche et al., 1996; Chappell and Webb, 2010), the interchangeable plastocyanin (copper binding) and cytochrome *c*_553_ (Fe binding) genes (*petE, Tery_2563* and *petJ,Tery_2561*, respectively) (Wood, 1978; Peers and Price, 2006; De la Cerda et al., 2007), genes for the Fe storage proteins bacterioferritin (*bfr, Tery_2787*) and ferritin (*ftn, Tery_4282*) (Keren et al., 2004; Marchetti et al., 2009), the ferric uptake regulators (*fur1, Tery_1958*; *fur2- Tery_3404* and *fur3, Tery_1953*) (González et al., 2012, 2016), the Fe binding nitrogenase NifH subunit (*nifH, Tery_4136*) (Shi et al., 2007; Richier et al., 2012; Snow et al., 2015a) and fructose bisphosphate aldolase class II (*fbaA, Tery_1687*) (Snow et al., 2015a).

**FIGURE 4:**
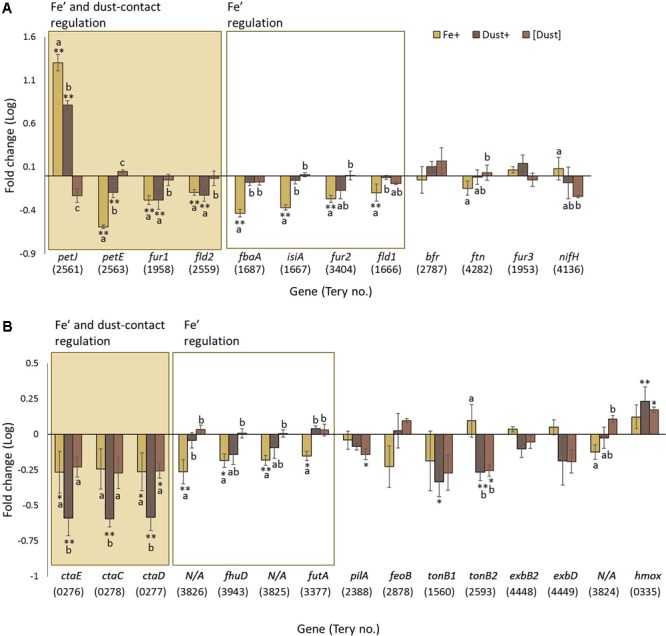
Transcription changes in genes of interest across the experimental treatments. Gene regulation (log fold change) in Fe+ (yellow), Dust+ (dark brown) and [Dust] (light brown) treatments all compared to Fe–, is presented for **(A)** genes expected to be regulated by Fe and **(B)** genes predicted to be involved in Fe utilization. Significantly differentially transcribed genes (Wald test, *P* < 0.05) due to both direct dust additions (Dust+ vs. [Dust]) and Fe’ availability (Fe+ vs. Fe–) (shaded box) or replete Fe’ only (white box) are plotted in order of highest to lowest fold change. Differences to Fe– are depicted with single (^∗^) or double (^∗∗^) stars for *P* < 0.05 and *P* < 0.01, respectively. Similarities between treatments Fe+, Dust+ and [Dust] are indicated as identical letter groups (a–c).

Four of these twelve genes, *petJ, petE, fur1* and *fld2*, show significant regulation both in response to direct dust addition (Dust+ compared to [Dust]) and increased availability of dissolved Fe (Fe’) (Fe+ compared to Fe-) while a further four genes, *fbaA, isiA, fur2* and *fld1*, were only significantly regulated through variable Fe’ (**Figure 4A**). Genes *bfr, ftn, fur3* and *nifH* were not found to be differentially regulated by any experimental condition.

### Molecular Signatures of Fe Utilization

Analysis of a further set of genes (Supplementary Table S2) was targeted to better understand mechanisms of Fe acquisition under the differing growth conditions. Suggested mechanisms for Fe reduction by cyanobacteria include the transfer of electrons to extracellular Fe^3+^ through the plasma-membrane-located alternative respiratory terminal oxidase (ARTO) (Kranzler et al., 2014) and potentially electrically conductive pili (Lamb et al., 2014). Genes *ctaC* (*Tery_0278*), *ctaD* (*Tery_0277*), and *ctaE* (*Tery_0276*) encoding subunits of ARTO displayed elevated transcription in Fe- compared to Fe+ and [Dust] compared to Dust+ (**Figure 4B**) revealing regulation by Fe’ and dust additions within the vicinity of the cells. Interestingly, transcripts of these genes were significantly downregulated in Dust+ compared to all other treatments. The *pilA* (*Tery_2388*) gene encoding the pili protein A was not differentially transcribed as a function of Fe’ (Fe+ compared to Fe-) and although transcription was reduced by dust (compared to Fe-), this was only significant when dust was separated from the cells (**Figure 4B**).

Well-characterized cyanobacterial Fe uptake pathways include the FutABC and FeoABC systems for transport of ferric (Fe^3+^) and ferrous (Fe^2+^) iron, respectively, across the inner membrane (Kammler et al., 1993; Katoh et al., 2001a,b). The *futA* (*Tery_3377*) gene, annotated as *idiA* (iron-deficiency induced) and encoding the Fe-binding subunit of the Fut Fe^3+^ transporter (Polyviou et al., unpublished), was downregulated in response to increased Fe’ (**Figure 4B**). Similarly, the transcript abundance of *feoB* (*Tery_2878*) was reduced in Fe+ cultures compared to the other treatments although the difference was not statistically significant.

Translocation of organically complexed Fe to the periplasm in cyanobacteria happens through TonB dependent transporters (TBDTs) at the outer membrane which seem to require energy transduction by TonB and ExbBD (Larsen et al., 1999; Brinkman and Larsen, 2008; Ollis et al., 2009; Ollis and Postle, 2012; Ollis et al., 2012), although ExbB and ExbD have also been previously linked to inorganic Fe uptake in *Synechocystis* sp. PCC 6803 (Jiang et al., 2015). Transport of organically complexed Fe across the inner membrane employs Fec/FhuD systems (Krewulak and Vogel, 2008; Stevanovic, 2015). The only *Trichodesmium* homolog to *fhu/fec* genes, *fhuD* (*Tery_3943*) (encoding for a periplasmic binding protein homolog), was downregulated by increased Fe’ and dust when added to the immediate cellular environment (**Figure 4B**). Homologs of *tonB* (*Tery_1560, Tery_2593*) and *exbBD* (*exbB: Tery_1868, Tery_4448; exbD: Tery_4449*) were not identified to be significantly differentially regulated, although transcription appeared higher in the Fe+ and Fe- treatments compared to the [Dust] and Dust+ treatments (with the exception of *Tery_1868* for which read counts were below the threshold for inclusion in the analysis) (**Figure 4B**).

Members of a previously identified putative siderophore production and uptake pathway, proteins *Tery_3824–3826* (Snow et al., 2015a) were observed to be significantly downregulated under increased Fe’ (significant difference for *Tery_3825 and Tery_3826*) (**Figure 4B**). Finally, transcription of the heme oxygenase homolog (*hmox, Tery_0335*), involved in extraction of heme-bound Fe was stimulated by the dust treatments compared to Fe+ and Fe- irrespective of the separation from the cells using the DT (**Figure 4B**). Some indication of increased transcript abundance due to increased Fe’ was also observed although this was not statistically significant.

### Strongly Differentially Regulated Uncharacterized Genes

**FIGURE 5:**
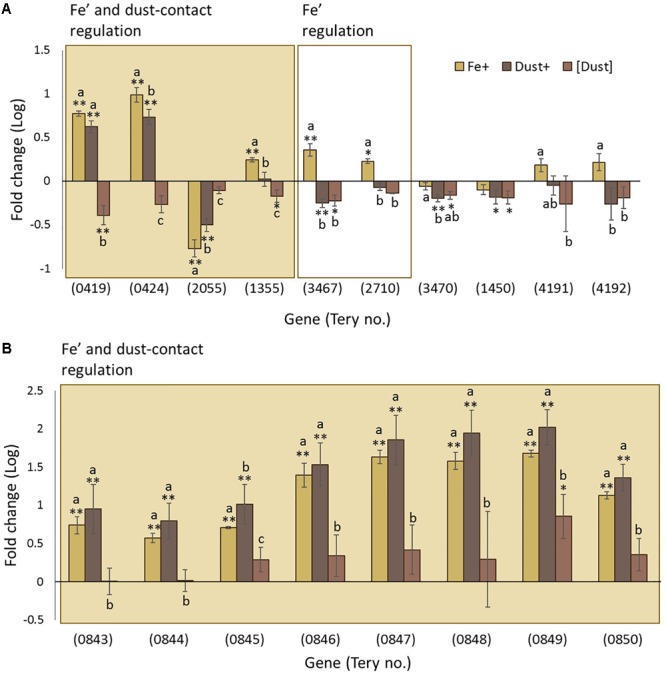
Transcription changes in uncharacterized genes across the experimental treatments. Regulation of **(A)** Haemolysin-Type Calcium Binding (HTCaB)-like genes and **(B)** genes of the *Tery_0843–0850* cluster are shown as log fold change for Fe+ (yellow), Dust+ (dark brown) and [Dust] (light brown) treatments all compared to Fe–. Significantly differentially transcribed genes (Wald test, *P* < 0.05) due to both direct dust additions (Dust+ vs. [Dust]) and Fe’ availability (Fe+ vs. Fe–) (shaded box) or replete Fe’ only (white box) are plotted in order of highest to lowest fold change **(A)** or order in the genome **(B)**. Differences to the Fe– are depicted with single (^∗^) or double (^∗∗^) stars for *P* < 0.05 and *P* < 0.01, respectively, while similarities between treatments Fe+, Dust+ and [Dust] are indicated as identical letter groups (a–c).

One third of the differentially regulated genes in the analysis were not GO annotated. Amongst them was a series of genes encoding for proteins with Haemolysin-Type Calcium Binding (HTCaB)-like domains (Supplementary Table S3) members of which were identified amongst the most differentially regulated genes in this study. Of 10 genes annotated as having a HTCaB-like domain and identified as regulated by some experimental condition in our analysis, *Tery_0419, Tery_0424, Tery_2055* and *Tery_1355*, are regulated by Fe’ and when contact with dust is permitted and *Tery_3467* and *Tery_2710* by Fe’ only (**Figure 5A**). Bioinformatic analysis indicates that HTCaB genes include multiple CHRD domains (identified in chordin) expected to have an immunoglobulin-like β-barrel structure based on some similarity to superoxide dismutases (Hyvönen, 2003). Among these genes, *Tery_3467* which was regulated by Fe’ has been previously identified as the zinc binding alkaline phosphatase (APase) gene *phoA* (Orchard et al., 2003).

Also among the uncharacterized genes is a cluster which spans eight genes (*Tery_0843–Tery_0850*) all of which were strongly differentially regulated in response to Fe’ availability and dust addition to the direct cellular vicinity (**Figure 5B**) with five (*Tery_0850, Tery_0845, Tery_0846, Tery_0849, Tery_0847*) amongst the top twenty most differentially transcribed genes in the RNAseq analysis. Tery_0848 and Tery_0849 are annotated as cell surface proteins while Tery_0843 is recognized by the UniProtKB Automatic Annotation pipeline as a membrane protein (Supplementary Table S3). In addition, Tery_0844 is predicted to contain an iron-sulfur binding site and Tery_0845 belongs to the heme oxygenase superfamily. The fifth gene in this cluster (*Tery_0847*) encodes for MetE (5-methyltetrahydropteroyltriglutamate-homocysteine S-methyltransferase) which catalyzes the transfer of a methyl group to and from methionine (Supplementary Table S3).

### Regulatory DNA

**FIGURE 6:**
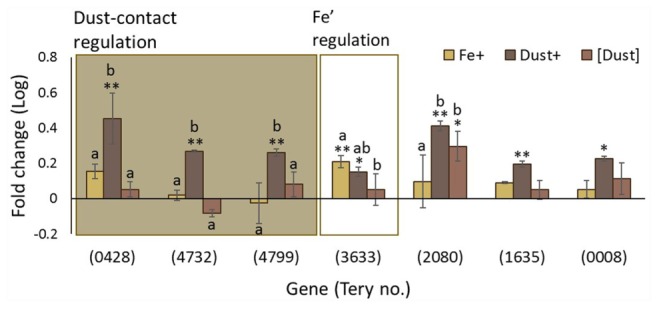
Changes in transcription of genes associated with non-protein coding DNA in *Trichodesmium*. Gene regulation (log fold change) in Fe+ (yellow), Dust+ (dark brown) and [Dust] (light brown) treatments all compared to Fe–. Group II intron host genes (as identified in Pfreundt et al., 2014) are significantly differentially transcribed (Wald test, *P* < 0.05) in response to dust in their immediate environment (Dust+ vs. [Dust]) (shaded box), or Fe’ availability (Fe+ vs. Fe–) (white box). Differences to the Fe- treatment are depicted with single (^∗^) or double (^∗∗^) stars for *P* < 0.05 and *P* < 0.01, respectively, while similarities between treatments Fe+, Dust+ and [Dust] are indicated as identical letter groups (a–c).

*Trichodesmium* has a large genome, with abundant non-protein coding regions, and has recently been shown to exploit sophisticated gene regulation (Pfreundt et al., 2014; Pfreundt and Hess, 2015; Walworth et al., 2015), as well as being polyploid (Sargent et al., 2016). The *Trichodesmium* genome harbors 17 group-II introns interrupting a total of 11 genes, 7 of which (*Tery_0428, Tery_4732, Tery_4799, Tery_3633, Tery_2080, Tery_1635, and Tery_0008*) are differentially regulated in one or more of the experimental treatments (**Figure 6**). Genes *Tery*_*0428, Tery_4732*, and *Tery_4799* are regulated only when dust is added directly to the cell environment, while *Tery_2080* is expressed more highly in both Dust+ and [Dust] compared to the Fe- and Fe+ treatments. Significant Fe’ regulation is only observed for *Tery_3633.*

## Discussion

Using cultures of filamentous *Trichodesmium erythraeum* IMS101 we show that these organisms can utilize Saharan desert dust as its sole source of Fe. However, this appeared to only be the case under conditions where direct physical contact was possible, as when dust was separated from the cells using dialysis tubing, both growth rate and photosynthetic efficiency were significantly reduced (**Figure 1**). Moreover, growth rates within media pre-incubated abiotically with dust, irrespective of whether it was constrained within or external to dialysis tubing, were indistinguishable to low Fe cultures and lower than Fe amended cultures (Supplementary Figure S2). In contrast, dust added directly to the growth media with the cells present supported similar growth rates and photosynthetic physiology to cells grown through the addition of dissolved Fe to EDTA buffered media (i.e., increased Fe’), all suggesting that direct contact between cells and dust particles or colloids might be necessary to facilitate Fe-acquisition from dust. Microscopic determination of such contact was not attempted here and therefore whether such contact is transient or if robust, long-term adhesion of particles to filaments occurs cannot be specified. Disentangling the nature of cell-to-particle contact can be challenging but it is perhaps interesting to consider future studies directed towards the identification of cell features which facilitate this interaction.

Cell surface processes occurring in environmentally collected *Trichodesmium* of the puff colony morphology have previously been suggested to enhance dissolution of Fe from dust (Rubin et al., 2011). The efficiency of colonies specifically, as opposed to filaments, for Fe acquisition was suggested to be attributed to specific features of the *Trichodesmium* colonies and the associated microbial consortia (Rubin et al., 2011). Our observations suggest that, in addition, processes which can be undertaken by filamentous *Trichodesmium*, potentially including any associated bacterial consortia, may also be relevant. Although not investigated in this study, microbial communities with some similarity to those found in the natural environment are associated with *Trichodesmium* cultures and can potentially influence the physiological response of *Trichodesmium* to the various conditions relating to the acquisition of Fe and ROS detoxification (Lee et al., 2017).

Potential molecular processes underlining the observed physiological differences between treatments were identified through RNA sequencing analysis on samples acquired at the end of the growth experiment. Such an analysis only provides a snapshot of the molecular response to the experimental conditions which is also likely to change temporally over the course of the experiment. However, clustering of transcription patterns in our experiment indicated that dust added cultures had a similar transcriptomic profile to cells grown replete with Fe’, only when contact of the cells with dust was permitted (**Figure 3**). In contrast, when dust was separated from cells, transcription patterns were more similar to that of Fe-deficient cultures. These observations further suggest that filaments can access Fe from dust by employing processes acting at the cell surface, although it should be noted that the pore size of the dialysis tubing, 8 kDa MWCO, will also prevent colloids from passing. Some of the differences between the two dust treatments could thus have resulted from a proportion of any bioavailable Fe released from the dust passing through a colloidal phase before being taken up by *Trichodesmium* and thus we cannot fully differentiate between likely cell surface interaction direct with the particulate dust or smaller colloids which may be derived from it. However, results from the abiotic control experiment (Supplementary Figure S2), argue that ‘dissolution’ of either soluble or colloidal Fe would appear to be insufficient to augment growth rates within our experimental conditions.

Pertinently, transcriptional patterns caused by Fe’ and dust were also distinct between all conditions (**Figure 3B**), suggesting complex transcriptional regulation, likely associated with some combination of the different nature of Fe in the treatments, the overall amounts of available Fe provided in each case, and/or the supply of additional nutrients from dust. Indeed, the differential transcription profiles between the dialysis isolated dust treatment and both the +Fe and -Fe treatments demonstrate that the presence of the former also had an observable biological effect, suggestive of the passage of some soluble constituents other than Fe across the membrane.

An analysis of 12 genes with known Fe regulation patterns was performed to assess the relative Fe status of the cultures (**Figure 4A**). The majority showed the predicted pattern of regulation by differential Fe’ availability and 4 were similarly regulated when dust was directly accessible to the cells. Since this was not the case after physical separation of dust from the cells, we suggest that specific cell surface processes are likely to be involved in acquiring Fe from dust. In this case, the improved physiology when dust was within the cell environment can be explained through additional Fe acquisition in this treatment. Some Fe biomarker genes did not show the expected pattern of regulation in dust amended cultures. While this could be due to the cells experiencing different severities of Fe stress when grown with replete Fe’ or dust, it may also reflect a specific response to Fe depending on its source and this may impact the interpretation of the transcription of certain Fe stress biomarker genes (Chappell and Webb, 2010; Richier et al., 2012; Snow et al., 2015a; Spungin et al., 2016).

The mechanisms by which *Trichodesmium* transports Fe across the cell membrane can potentially be inferred through homology to other know Fe transporters and omics responses to Fe replete versus deplete conditions (Shi et al., 2007; Chappell et al., 2012; Snow et al., 2015a). Genes such as *futA* and *feoB* encoding members of these transport systems showed the expected downregulation in response to replete Fe’, but not due to dust (**Figure 4B**) likely reflecting differences in the response of this organism depending on the source of Fe. The mechanisms by which *Trichodesmium* accesses particulate, or colloidal, Fe and transports Fe across the outer membrane are unknown. The only characterized system in cyanobacteria involves TBDTs powered by the TonB and ExbBD proteins. Homologs to these genes were not regulated by Fe in our study (**Figure 4B**) suggesting they might be regulated by other factors, or were possibly not required for accessing the types of Fe used in this study (Schauer et al., 2008).

A molecular level understanding of Fe reduction prior to its uptake in cyanobacteria is also lacking. The cell membrane localized ARTO of *Synechocystis* sp. PCC 6803, was recently indicated to use Fe^3+^ as an electron acceptor, reducing it to Fe^2+^ in the periplasmic space prior to its uptake by FeoAB (Kranzler et al., 2014). Downregulation by replete Fe’ and direct dust addition to the cells as observed here (**Figure 4B**) for *Trichodesmium* supports possible involvement of the complex, not only in reduction and subsequent acquisition of Fe’, but also of Fe provided by dust particles. It is of interest that the largest reduction in expression of subunits of ARTO was observed when cells were in the direct vicinity of dust particles. While this may reflect summative influences of increased Fe availability and the presence of dust, it suggests that filaments may have a dramatically reduced requirement for any ARTO mediated Fe uptake when exposed to dust. As the depleted, oxidized nature of Fe in the marine environment could render ARTO-mediated Fe reduction a limiting step to Fe uptake and consequently to *Trichodesmium* growth, further analysis is required to fully elucidate the importance of this pathway. Finally, a possible siderophore production/utilization pathway (Tery_3823–3826) (Snow et al., 2015a) was also regulated by Fe’ availability (**Figure 4B**) but further evidence is required to characterize its involvement in Fe utilization by *Trichodesmium*.

With a large fraction of *Trichodesmium* genes either mis-annotated or annotated as encoding hypothetical proteins of unknown function, it is likely that a wealth of information regarding its physiological adaptations to Fe depletion are yet to be determined. However, based on strong differential transcription, we identify a protein class not previously recognized to be Fe regulated, and a gene cluster encoding proteins with putative extracellular or outer membrane functions. The former includes HTCaB region domain proteins (**Figure 5A**) which, as far as we can ascertain, have not been studied previously in *Trichodesmium*. HTCaB domains occur in tandem repeats in proteins that can form a parallel β roll structure (Baumann et al., 1993; Lilie et al., 2000) and are exported from the cell to function as haemolysins, cyclolysins, leukotoxins and metallopeptidases (Boehm et al., 1990; Duong et al., 1992; Rose et al., 1995). Further they may have adhesive properties (Sánchez-Magraner et al., 2007) and roles in motility (Brahamsha and Haselkorn, 1996; Hoiczyk and Baumeister, 1997; Pitta et al., 1997). These characteristics, alongside the differential regulation observed here (**Figure 5A**) suggest an important function of the proteins in Fe metabolism and a putative role in attachment to particles or mobilization of Fe from the dust. Our data also indicates a cluster of eight consecutive genes (*Tery_0843–Tery_0850*, Supplementary Table S3), which are strongly responsive to direct dust additions and also regulated by Fe’ availability. The encoded uncharacterised proteins have features associating them with the cell membranes/surface and possibly the degradation of Fe-containing compounds like heme (**Figure 5B**).

Lastly, transcriptional patterns suggest a significant involvement of non-protein coding DNA in the Fe’ and dust response of *Trichodesmium*. The *Trichodesmium* genome has a large non-coding content (40% against the cyanobacterial average of 15%) with the most non-coding DNA transcription start sites (TSS) of currently analyzed bacterial species (Pfreundt et al., 2014; Pfreundt and Hess, 2015). That the non-protein coding fraction of the genome is maintained in the environment (Walworth et al., 2015), leads to the conclusion that regulatory RNA is linked to the organisms’ lifestyle, possibly its cohabitation with other microorganisms and the nutrient fluctuations it encounters. In support of this, we observe that group II intron host genes were differentially regulated in response to Fe’ and/or dust (**Figure 6**). Based on these results we suggest that *Trichodesmium’s* non-coding DNA could be involved in facilitating the regulatory complexity required in a fluctuating environment with ephemeral Fe supplies.

## Conclusion

We present evidence that cell-to-particle interaction may be a key component of Fe acquisition and the broader ecophysiological functioning of *Trichodesmium*. Cells grown in the direct vicinity of dust displayed unique physiological and molecular characteristics that contrasted with those only having access to soluble species released from the same substrate and from those grown under Fe stress and Fe replete conditions through manipulation of Fe’ availability. In addition to providing further evidence that substrate specific responses might influence the bioavailability of Fe to *Trichodesmium in situ*, these results further suggest that variability in substrate might need to be directly considered when using Fe uptake transporters as *in situ* markers of Fe limitation. Indeed, the generated transcriptomic profiles could potentially be used for the identification of specific traits linked to the responses of *Trichodesmium* to differing modes of Fe supply, a stepping stone in better understanding the apparent niche success of this organism within Fe enriched areas of the more generally anemic open oceans.

## Author Contributions

DP, AH, CM, and TB conceived and planned the experiments. DP carried out the experiments. DP and AB analyzed the data. DP, AB, AH, JR, CM, and TB interpreted the data and wrote the manuscript.

## Conflict of Interest Statement

The authors declare that the research was conducted in the absence of any commercial or financial relationships that could be construed as a potential conflict of interest.
