# Supplementary material for: Desert Dust as a Source of Iron to the Globally Important Diazotroph Trichodesmium
Source: Front Microbiol. 2018 Jan 17;8:2683. doi: 10.3389/fmicb.2017.02683 (PMC5776111; doi:10.3389/fmicb.2017.02683)
Supplement: Supplementary file 1 [file Data_Sheet_1.docx]

Supplementary Material

**Desert Dust as a Source of Iron to the Globally Important Diazotroph *Trichodesmium***

**Despo Polyviou^1*^, Alison J. Baylay^1^, Andrew Hitchcock^2^, Julie Robidart^3^, C. Mark Moore^1^ and Thomas S. Bibby^1^**

^1^ Ocean and Earth Sciences, University of Southampton, Waterfront Campus, European Way, Southampton SO14 3ZH, UK.

^2^ Department of Molecular Biology and Biotechnology, University of Sheffield, Firth Court, Western Bank, Sheffield S10 2TN, UK.

^3^ Ocean Technology and Engineering, National Oceanography Centre, European Way, Southampton SO14 3ZH, UK

* **Correspondence:**

Despo Polyviou

d.polyviou@noc.soton.ac.uk

**Keywords: *Trichodesmium*, iron, dust, nitrogen fixation, cyanobacteria**

**Supplementary Tables**

**Table S1:** Genes known to be regulated by Fe or identified as putative biomarkers of Fe limitation.

| **Tery no.** | **Gene name** | **Function** | **References** |
| --- | --- | --- | --- |
| *Tery_1667* | *isiA* | chlorophyll-binding antenna | Bibby et al. 2001; Shi et al. 2007; Richier et al. 2012, Snow et al. 2015 |
| *Tery_1666* | *fld1* | flavodoxin | LaRoche et al. 1996, Chappell and Webb 2010 |
| *Tery_2559* | *fld2* | flavodoxin |  |
| *Tery_2563* | *petE* | plastocyanin | Wood 1978, Peers & Price 2006, De la Cerda et al. 2007 |
| *Tery_2561* | *petJ* | cytochrome *c*_553_ |  |
| *Tery_2787* | *bfr* | bacterioferritin | Keren et al. 2004, Marchetti et al 2009 |
| *Tery_4282* | *ftn* | ferritin |  |
| *Tery_1958* | *fur1* | ferric uptake regulator | González et al., 2012, 2016 |
| *Tery_3404* | *fur2* | ferric uptake regulator |  |
| *Tery_1953* | *fur3* | ferric uptake regulator |  |
| *Tery_4136* | *nifH* | nitrogenase subunit | Shi et al. 2007, Richier et al 2012, Snow et al 2015 |
| *Tery_1687* | *fbaA* | fructose bisphosphate aldolase class II | Snow et al. 2015 |

**Table S2:** Identified genes with a known or predicted function in Fe acquisition.

| **Tery no.** | **Gene name** | **Function** | **References** |
| --- | --- | --- | --- |
| *Tery_0276* | *ctaE* | ARTO subunit | (Kranzler et al. 2014; Snow et al. 2015) |
| *Tery_0277* | *ctaD* | ARTO subunit |  |
| *Tery_0278* | *ctaC* | ARTO subunit |  |
| *Tery_3824* |  | putative siderophore uptake | (Snow et al. 2015) |
| *Tery_3825* |  | putative siderophore uptake |  |
| *Tery_3826* |  | putative siderophore uptake |  |
| *Tery_3943* | *fhuD* | siderophore transporter binding protein | (Krewulak & Vogel 2008; Chappell & Webb 2010; Stevanovic 2015) |
| *Tery_3377* | *futA2* | Fe^3+^ transporter binding protein | (Chappell & Webb 2010; Stevanovic 2015, Kranzler et al. 2014) |
| *Tery_2388* | *pilA* | major pilin protein | (Lamb et al. 2014; Snow et al. 2015) |
| *Tery_2878* | *feoB* | ferrous iron transporter protein B | (Kammler et al. 1993; Chappell & Webb 2010) |
| *Tery_1560* | *tonB1* | transport protein | (Hopkinson & Barbeau 2012; Chappell & Webb 2010) |
| *Tery_2593* | *tonB2* | transport protein |  |
| *Tery_4448* | *exbB2* | transport protein |  |
| *Tery_4449* | *exbD* | transport protein |  |
| *Tery_0335* | *hmox* | Heme oxygenase | (Runyen-Janecky 2013) |

**Table S3: The *Trichodesmium* Tery 0843- 0850 cluster components.** Predicted conserved domains, subcellular localization or metal binding sites as identified by the UniProtKB Automatic Annotation pipeline are indicated.

| **Tery no** | **Annotation** | **Size (bp)** | **Protein features** |
| --- | --- | --- | --- |
| Tery_0843 | hypothetical protein | 609 | 5 alpha-helical transmembrane regions |
| Tery_0844 | putative iron-sulfur cluster-binding protein | 876 | [2Fe-2S] cluster binding site |
| Tery_0845 | TENA/THI-4 protein | 705 | Heme oxidase superfamily |
| Tery_0846 | hypothetical protein | 459 | - |
| Tery_0847 | 5-methyltetrahydropteroyltriglutamate/homocysteine S-methyltransferase | 2235 | methionine synthesis  3 zinc-binding regions |
| Tery_0848 | cell surface protein | 183 | - |
| Tery_0849 | cell surface protein | 573 | - |
| Tery_0850 | hypothetical protein | 141 | - |

## Supplementary Figures


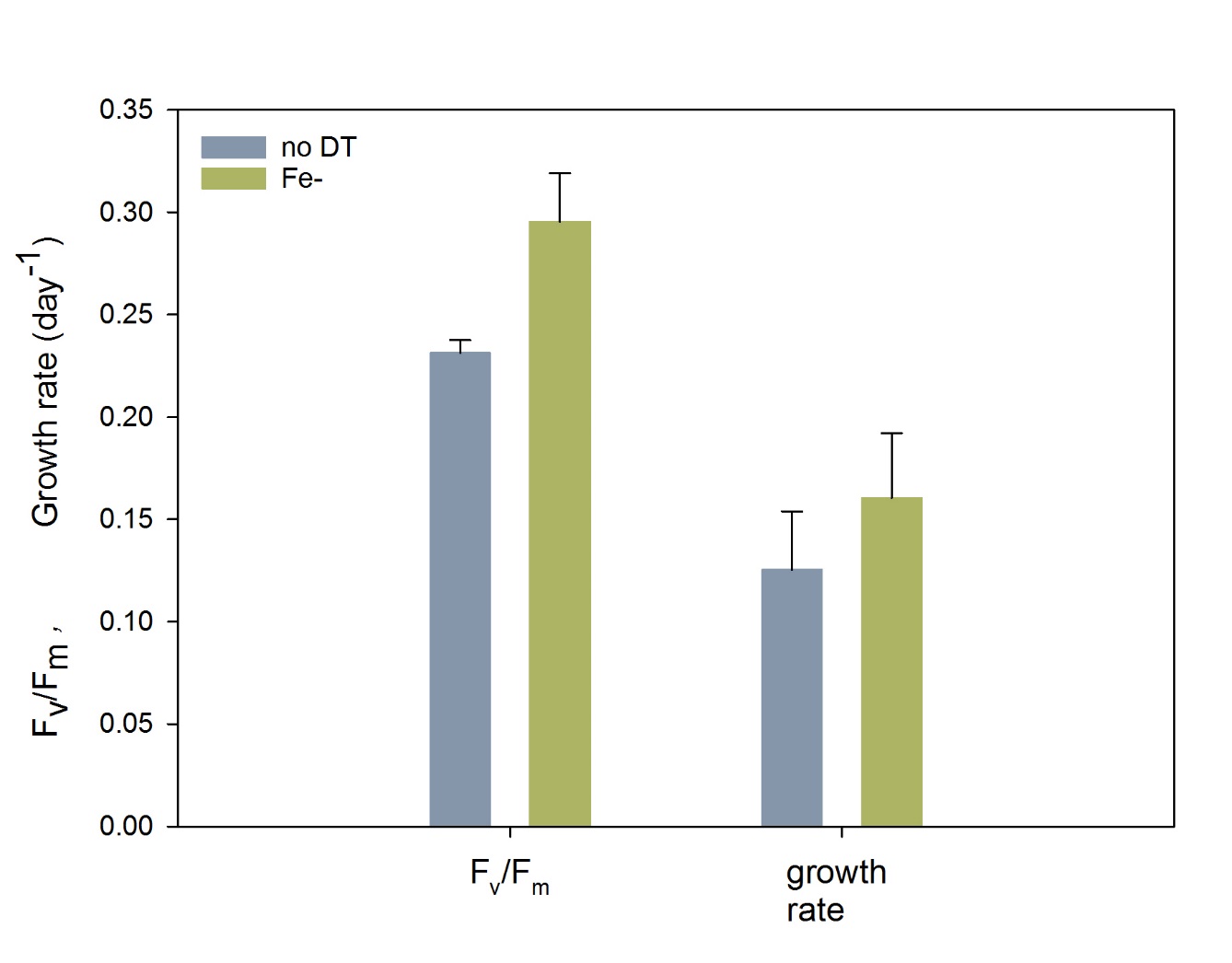


**Figure S1:** **Assessment of potential dialysis tubing effects when included in the experiment.** Comparison of the no dialysis tubing (no DT) control treatment to the equivalent Fe- treatment with DT included (Fe-) indicates that photosynthetic efficiency (F*v*/F*m*) measured on the final day of the experiment and growth rates did not differ significantly between the two treatments (GLM and Tukey test). Error bars represent standard deviations from the mean of three biological replicates.


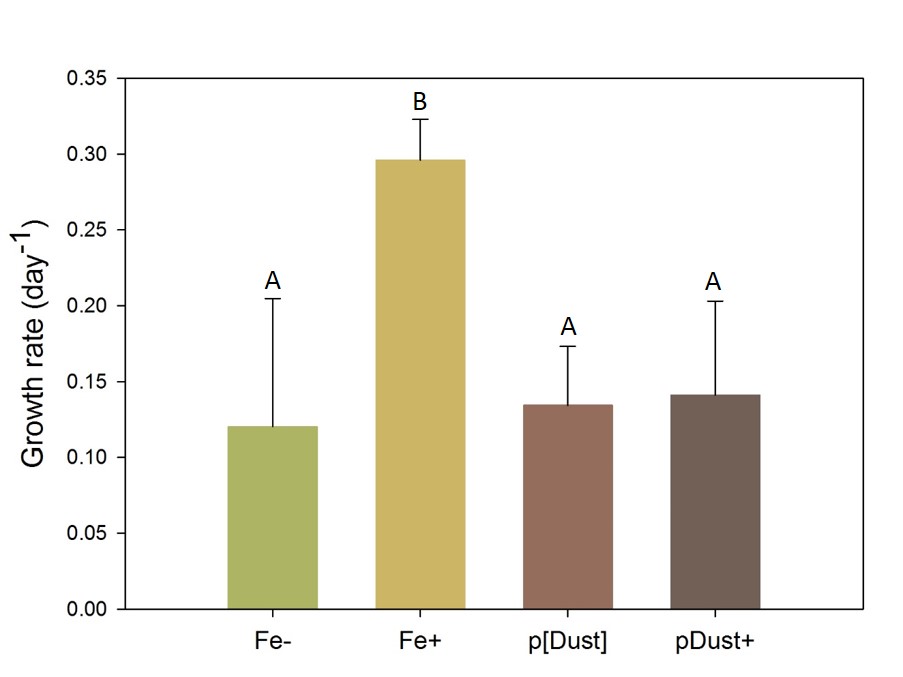


**Figure S2: Abiotic dissolution control experiment.** Conditions during the abiotic dissolution phase were identical to the original experiment but without inoculation with *Trichodesmium* cells. Following a period of 14 days all treatments were filtered through 0.22 μm sterile filters and the corresponding mYBC-II was then inoculated with equal concentrations of *Trichodesmium* cells. Increased growth rates were identified in FeCl_3_-EDTA added cultures (Fe+, yellow) compared to all other treatments. Cultures whereby the media was previously incubated with dust inside (p[Dust], light brown) or outside (pDust+, dark brown) the DT had similarly reduced growth rates, not significantly different to Fe deprived cultures (Fe-, green). Distinct letter groups (A and B) mark significant differences (GLM and Tukey test, P < 0.05) between treatments while error bars represent standard deviations from the mean of three biological replicates.


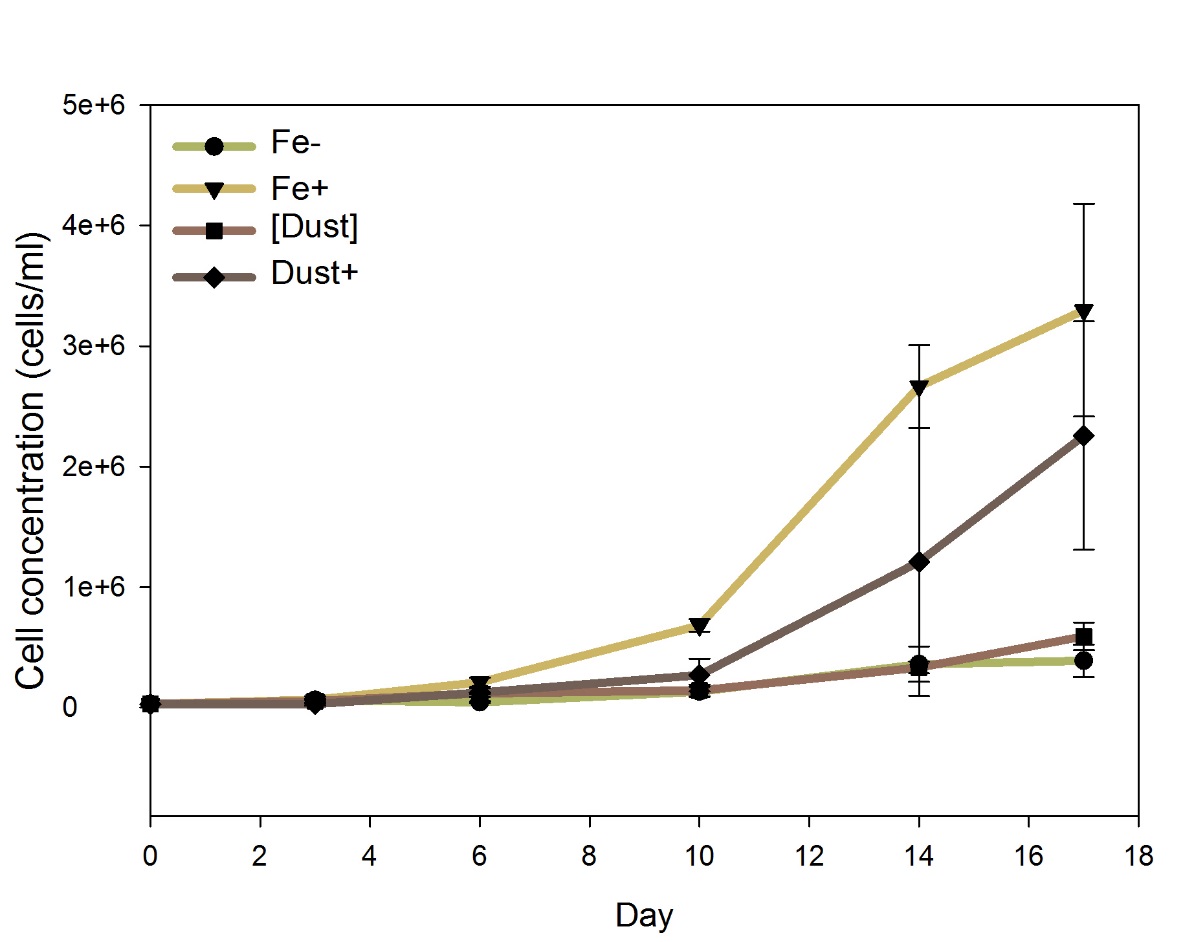


**Figure S3**: **Growth curves recorded over 17 days are presented for each of the experimental treatments.** Growth was stimulated by additions of FeCl_3_-EDTA (Fe+, yellow) and dust when in the direct cellular environment (Dust+, dark brown). Minimal growth was recorded both when dust was separated from the cells ([Dust], light brown) and when no Fe was added to the media (Fe-, green). Error bars represent standard deviations from the mean of three biological replicates.

**
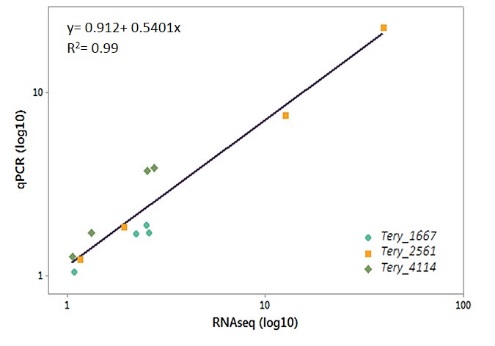
**

**Figure S4:** **Validation of the RNAseq results using quantitative RT-PCR (qPCR).** Regression analysis indicated a correspondence of calculated relative fold change (RFC) in expression between RNAseq analysis and qPCR for genes *Tery_1667* (*isiA*), *Tery_2561* (*cyt c_553_*) and *Tery_4114*. (nif-like gene). RFC was calculated by normalization to the lowest expression across the four treatments (Fe-, Fe+, [Dust], Dust+).


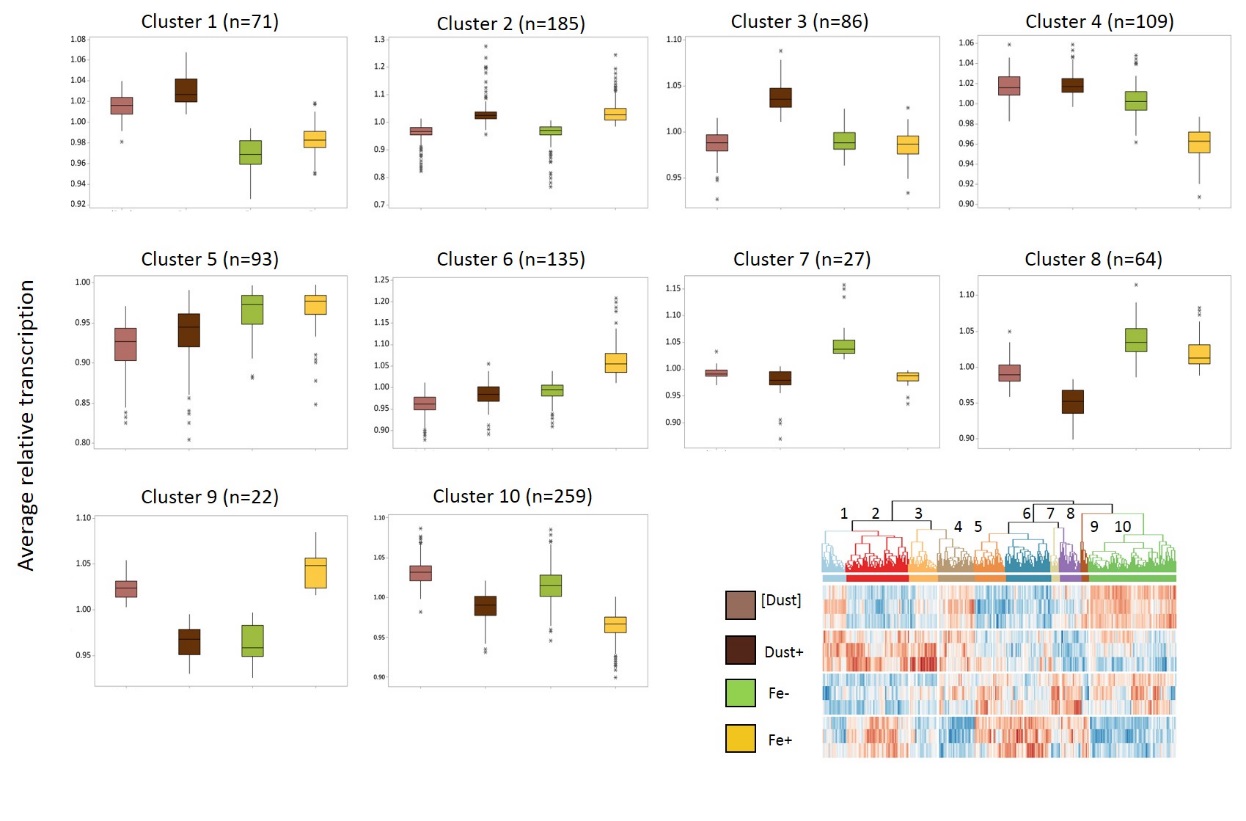


**Figure S5:** **Gene transcription profiles of 10 distinct groups of genes.** Hierarchical clustering (WPGMA method with 1-PPMCC as the distance metric) following calculation of the Pearson product moment correlation coefficient (PPMCC) for each pair of genes, was used to group different transcription patterns presented in a heatmap (bottom right). Boxplots indicate transcription across treatments for each cluster (average transcription for each treatment corrected to the average across all treatments) and the numbers of genes in each cluster are highlighted.

**References**

Bibby, T., Nield, J., and Barber, J. (2001). Iron deficiency induces the formation of an antenna ring around trimeric photosystem I in cyanobacteria. *Nature* 412, 743–745. doi: 10.1038/35089098

Chappell, P. D., and Webb, E. (2010). A molecular assessment of the iron stress response in the two phylogenetic clades of *Trichodesmium*. *Environ. Microbiol*. 12, 13–27. doi: 10.1111/j.1462-2920.2009.02026.x

De la Cerda, B., Castielli, O., Durán, R. V., Navarro, J. A., Hervás, M., and De la Rosa, M. A. (2007). A proteomic approach to iron and copper homeostasis in cyanobacteria. *Brief. Funct. Genomic Proteomic* 6, 322–329. doi: 10.1093/bfgp/elm030

González, A., Bes, M. T., Peleato, M. L., and Fillat, M. F. (2016). Expanding the role of FurA as essential global regulator in cyanobacteria. *PLOS ONE* 11:e0151384. doi: 10.1371/journal.pone.0151384

González, A., Bes, M. T., Valladares, A., Peleato, M. L., and Fillat, M. F. (2012). FurA is the master regulator of iron homeostasis and modulates the expression of tetrapyrrole biosynthesis genes in Anabaena sp. PCC 7120. *Environ. Microbiol*. 14, 3175–3187. doi: 10.1111/j.1462-2920.2012.02897.x

Hopkinson, B. M., and Barbeau, K. A. (2012). Iron transporters in marine prokaryotic genomes and metagenomes. *Environ. Microbiol*. 14, 114–128. doi: 10.1111/j.1462-2920.2011.02539.x

Kammler, M., Schön, C., and Hantke, K. (1993). Characterization of the ferrous iron uptake system of *Escherichia coli*. *J. Bacteriol*. 175, 6212–6219. doi: 10.1128/ jb.175.19.6212-6219.1993

Keren, N., Aurora, R., and Pakrasi, H. B. (2004). Critical roles of bacterioferritins in iron storage and proliferation of cyanobacteria. *Plant Physiol*. 135, 1666–1673. doi: 10.1104/pp.104.042770

Kranzler, C., Lis, H., Finkel, O. M., Schmetterer, G., Shaked, Y., and Keren, N. (2014). Coordinated transporter activity shapes high-affinity iron acquisition in cyanobacteria. *ISME J*. 8, 409–417. doi: 10.1038/ismej.2013.161

Krewulak, K. D., and Vogel, H. J. (2008). Structural biology of bacterial iron uptake. *Biochim. Biophys. Acta* 1778, 1781–1804. doi: 10.1016/j.bbamem.2007.07.026

Lamb, J. J., Hill, R. E., Eaton-Rye, J. J., and Hohmann-Marriott, M. F. (2014). Functional role of PilA in iron acquisition in the Cyanobacterium *Synechocystis* sp. PCC 6803. *PLOS ONE* 9:e105761. doi: 10.1371/journal.pone.0105761

LaRoche, J., Boyd, P. W., McKay, R. M. L., and Geider, R. J. (1996). Flavodoxin as an *in situ* marker for iron stress in phytoplankton. *Nature* 382, 802–805. doi: 10.1038/382802a0

Marchetti, A., Parker, M. S., Moccia, L. P., Lin, E. O., Arrieta, A. L., Ribalet, F., et al. (2009). Ferritin is used for iron storage in bloom-forming marine pennate diatoms. *Nature* 457, 467–470. doi: 10.1038/nature07539

Peers, G., and Price, N. M. (2006). Copper-containing plastocyanin used for electron transport by an oceanic diatom. *Nature* 441, 341–344. doi: 10.1038/ nature04630

Richier, S., Macey, A. I., Pratt, N. J., Honey, D. J., Moore, C. M., and Bibby, T. S. (2012). Abundances of iron-binding photosynthetic and nitrogen-fixing proteins of *Trichodesmium* both in culture and in situ from the North Atlantic. *PLOS ONE* 7:e35571. doi: 10.1371/journal.pone.0035571

Runyen-Janecky, Laura J. (2013). Role and regulation of heme iron acquisition in gram-negative pathogens. *Front. Cell Infect. Microbiol*. 3. doi: 10.3389/fcimb.2013.00055

Shi, T., Sun, Y., and Falkowski, P. G. (2007). Effects of iron limitation on the expression of metabolic genes in the marine cyanobacterium *Trichodesmium erythraeum* IMS101. *Environ. Microbiol*. 9, 2945–2956. doi: 10.1111/j.1462- 2920.2007.01406.x

Stevanovic, M. (2015). The Putative Siderophore-Dependent Iron Transport Network in *Anabaena* sp. PCC 7120. Frankfurt: Universitäts bibliothek Johann Christian Senckenberg.

Snow, J. T., Polyviou, D., Skipp, P., Chrismas, N. A. M., Hitchcock, A., Geider, R., et al. (2015a). Quantifying integrated proteomic responses to iron stress in the globally important marine diazotroph *Trichodesmium*. *PLOS ONE* 10:e0142626. doi: 10.1371/journal.pone.0142626

Wood, P. M. (1978). Interchangeable copper and iron proteins in algal photosynthesis. Studies on plastocyanin and cytochrome c-552 in *Chlamydomonas. Eur*. *J. Biochem*. 87, 9–19. doi: 10.1111/j.1432-1033.1978.tb12346.x
